# Amphetamine and pseudoephedrine cross-tolerance measured by c-Fos protein expression in brains of chronically treated rats

**DOI:** 10.1186/1471-2202-9-99

**Published:** 2008-10-06

**Authors:** Nootchanart Ruksee, Walaiporn Tongjaroenbuangam, Stefano O Casalotti, Piyarat Govitrapong

**Affiliations:** 1 Neuro-Behavioral Biology Center, Institute of Science and Technology for, Research and Development, Mahidol University, Salaya, Nakornpathom, 73170, Thailand; 2 Faculty of Lifelong Learning, Birkbeck, University of London, Malet Street, London, WC1E 7HX, UK; 3 UCL Ear Institute, University College London, 332 gray's Inn Road, London, WC1X 8EE, UK; 4 Center for Neuroscience and Department of Pharmacology, Faculty of Science, Mahidol University, Rama 6 Road, Bangkok, 10400, Thailand

## Abstract

**Background:**

Pseudoephedrine is a drug commonly prescribed as a nasal decongestant and bronchodilator and is also freely available in cold remedies and medications. The structural and pharmacological similarity of pseudoephedrine to amphetamine has led to evaluation of its psychomotor stimulant properties within the central nervous system. Previous investigations have shown that the acute responses to pseudoephedrine were similar to those of amphetamine and other psychostimulants.

**Results:**

This study examined the effect of chronic administration of pseudoephedrine in rat nucleus accumbens and striatum and identified three further similarities to amphetamine. (i) Chronic exposure to pseudoephedrine reduced the c-Fos response to acute pseudoephedrine treatment suggesting that pseudoephedrine induced tolerance in the animals. (ii) In animals chronically treated with amphetamine or pseudoephedrine the acute c-Fos response to pseudoephedrine and amphetamine was reduced respectively as compared to naïve animals indicating cross-tolerance for the two drugs. (iii)The known involvement of the dopamine system in the response to amphetamine and pseudoephedrine was further confirmed in this study by demonstrating that pseudoephedrine similarly to amphetamine, but with lower potency, inhibited [^3^H]dopamine uptake in synaptosomal preparations.

**Conclusion:**

This work has demonstrated further similarities of the effect of pseudoephedrine to those of amphetamine in brain areas known to be associated with drug addiction. The most significant result presented here is the cross tolerance effect of amphetamine and psudoephedrine. This suggests that both drugs induce similar mechanisms of action in the brain. Further studies are required to establish whether despite its considerable lower potency, pseudoephedrine could pose health and addiction risks in humans similar to that of known psychostimulants.

## Background

Pseudoephedrine is a drug commonly prescribed as a nasal decongestant and bronchodilator and is also available in over the counter cold remedy medications. The chemical structure of pseudoephedrine is similar to that of the psychostimulant amphetamine and both are classified as sympathomimetic drugs [1]. Numerous studies have documented the cellular effects of amphetamine which include increased dopamine release, inhibition of dopamine uptake, D1 and D2 dopamine receptor stimulation, cAMP changes, cAMP responsive element binding protein (CREB) activation, immediate-early gene expression and activation of other specific genes such as dynorphin[2].

Within the central nervous system, the nucleus accumbens and the striatum have been identified as important target areas of psychostimulants and opiates whereby repetitive activation of these areas through dopaminergic action is believed to play a major role in the establishment of drug dependence and withdrawal phenomena [3-5].

The expression of immediate-early genes in response to acute administration of potentially addictive drugs is of particular interest with respect to the mechanisms that may trigger dependence. Amphetamine can induce the expression of c-Fos and jun-B and zif:268. This ability to acutely induce immediate-early gene expression in the nucleus accumbens and striatum is now considered to be a general property of psychostimulant drugs [6-9]. The induction of c-fos by psychostimulants is believed to be mediated predominantly via the D1 receptors as demonstrated by the fact that the D1 specific antagonist SCH23390 can strongly inhibit amphetamine and cocaine-induced c-Fos expression and Fos-like immunoreactivity [6,10]. However, these findings do not preclude the involvement of other dopamine receptor types in the response to psychostimulants [8,11].

Work in our laboratory employing a drug substitution test indicated that pseudoephedrine elicited, in rats, similar internal cues to amphetamine [12]. Additionally, we demonstrated that pseudoephedrine induced Fos-like immunoreactivity in the nucleus accumbens and striatum regions in a time and concentration-dependent manner with maximal effect at 60 mg/kg 2 h after injection [13]. To further investigate the similarity of amphetamine's and pseudoephedrine's action we have analysed the acute c-Fos response to psuedoephedrine in rats chronically treated with pseudoephedrine or amphetamine. Additionally, we have compared the effect of pseudoephedrine and amphetamine on dopamine taken up into synaptosomes prepared from rat nucleus accumbens and striatum. The data further indicates that pseudoephedrine operates in a manner similar to amphetamine which may have implications for its over-the-counter use.

## Methods

### Animal treatments

All animal procedures were carried out in compliance with Mahidol University Code of Practice and the National Institutes of Health (USA) Guidelines for treatment of laboratory animals. Male Sprague-Dawley rats (between 200 and 250 g) were obtained from the National Animal Center, Mahidol University, Thailand, housed in groups of 6 and maintained on a 12 h light/dark cycle with free access to water and food. All animals were handled for at least 1 week before the experiment. In acute treatment, rats were injected intraperitoneally (i.p.) with pseudoephedrine (40 mg/kg, i.p.) or d-amphetamine (3 mg/kg, i.p.) and were killed 1.5 h after the drug injection. In chronic treatment, rats were injected twice a day (8.30 a.m. and 6.30 p.m.) for 8 days with pseudoephedrine (Sigma, Aldrich St. Louis USA) at increasing doses of 25, 30, 35, 40 mg/kg, i.p. on day 1, 2, 3 and 4–8 respectively. To study cross tolerance with amphetamine, rats were chronically injected twice a day (8.30 a.m. and 6.30 p.m.) for 8 days with d-amphetamine at increasing doses of 1, 2, 2.5, 3 mg/kg, i.p. on day 1, 2, 3 and 4–8 respectively. Rats were sacrificed on day 9, 1.5 h after the last injection of either d-pseudoephedrine (40 mg/kg, i.p.), d-amphetamine (3 mg/kg, i.p.) or saline. The final injection on day 9 was carried out at 8:30 a.m. as during the chronic treatment.

### Nuclear extract preparation

Under the stereodissecting microscope, the rat brains were cut coronally at the optic chiasma and 2 mm more frontally. From this section striatum and nucleus accumbens were manually dissected. The striatum was identified as the striated area below the corpus callosum while the nucleus accumbens as the area surrounding the anterior commisure which is easily identifiable as a small white area. The dissected tissues were used for nuclei isolation as previously described [14]. To obtain sufficient tissue, brain areas from 2–3 similarly treated animals were pooled and homogenized in 2 ml of chilled solution I (0.32 M sucrose, 3 mM MgCl2, 1 mM Hepes, pH 6.8) using a glass Teflon homogenizer. The crude homogenate was transferred to a new centrifuge tube and diluted with 1.2 ml of solution I and 0.44 ml of distilled water. The diluted homogenate was underlaid with 3 ml of solution I, and centrifuged at 1000 × g for 10 min. The pellet was harvested and resuspended in 5.33 ml of solution II (1.4 M sucrose, 1 mM MgCl2, 1 mM Hepes, pH 6.8), and centrifuged at 50,000 × g for 10 min in a fixed angle rotor. The nuclear pellet was harvested and resuspended in 18–20 μl of solution III (0.25 M sucrose, 1 mM MgCl2, 1 mM Hepes, pH 6.8). Protein concentration was determined by the method of Lowry [15] where bovine serum albumin was used as a standard.

### Western blot

Western blots were carried out as previously described [13]. Nuclear extract samples were mixed with an equal volume of loading buffer (0.1 M Tris (pH 6.8), 4% sodium dodecyl sulphate (w/v), 20% glycerol (w/v), 0.2 M of 1,4-Dithiothreitol (DTT) and 0.2% bromophenol blue (w/v), boiled for 5 min, electrophoresed on 10% discontinuous sodium dodecyl sulphate acrylamide gel and electroblotted to nitrocellulose membrane. Following transfer, the membrane was briefly washed in transfer buffer, and incubated in blocking solution (0.02 M Tris, pH 7.4, 0.15 M NaCl, 5% non-fat dried milk (w/v), 0.05% Tween 20 (v/v)) for 2 h at room temperature. The membrane was incubated with anti-Fos antibodies (Genosys Ltd., UK.) 1:500 in blocking solution for 1.5 h, washed 4 × 7 min with 50 ml of TBST solution (0.2 M Tris (pH 7.4), 1.5 M NaCl, 0.5% Tween 20 (v/v)) incubated with alkaline phosphatase-conjugated anti-sheep immunoglobulin antibody (Sigma, St. Louis USA) diluted 1:1000 in blocking solution for 1 h at room temperature and washed first 4 × 7 min with 50 ml of TBST solution and finally 7 min with 50 ml of TBS solution (0.2 M Tris (pH 7.4), 1.5 M NaCl). Bands were visualized after addition of Western Blue (Promega, USA) stabilized substrate for alkaline phosphatase. The staining of the specifically labeled 55 KDa c-Fos band was scanned on a flat bed scanner and quantified with the NIH software program.

### Synaptosome preparation

Male Sprague Dawley rats weighing 200–250 g were killed by decapitation and brains were rapidly removed and cooled on ice. The synaptosomes were prepared as previously described [16] with some modifications. The striatum and nucleus accumbens were dissected, weighed and homogenized using a glass teflon homogenizer in 20 volumes of 0.32 M sucrose, 0.12 mM KH2PO4 and 0.5 mM Na2HPO4 (pH 7.4) at 4°C. Homogenates were centrifuged at 900 × g for 10 min. The supernatant was further centrifuged at 11,000 × g for 20 min, The pellet was resuspended in an ice cold Krebs phosphate buffer containing 127.2 mM NaCl, 5 mM KCl, 1.3 mM MgSO4, 1.2 mM KH2PO4, 5 mM Na2HPO4, 1.25 mM CaCl2, 1 mM EDTA, 0.105 mM ascorbic acid, 1.05 μM pargyline and 11.1 mM glucose (pH 7.4).

### [^3^H]Dopamine uptake

Measurement of [^3^H]dopamine uptake into synaptosomes was carried out according to previously published methods [17,18]. Test drugs (50 μl of *d*-amphetamine or *d*-pseudoephedrine) followed by 50 μl [^3^H]dopamine (final concentration 1 nM) were added to synaptosomal suspensions (900 μl, final protein concentration of striatal and nucleus accumbens synaptosomes were 0.44 ± 0.01 mg/ml and 0.23 ± 0.01 mg/ml respectively) to give a final volume of 1 ml. The reaction mixture was incubated at 25°C for 5 min in a metabolic shaker water bath. Following incubation, the reaction mixture was rapidly filtered through glass-fiber filter paper (Whatman GF/B) by a vacuum pump and washed twice with 3 ml of Krebs phosphate buffer. The synaptosomes containing radioactive dopamine trapped on filter paper were counted by liquid scintillation spectrometry (Beckman LS 1801) in 5 ml of TritonX-100/Toluene base fluor (1:3) scintillation fluid. The active accumulation of [^3^H]dopamine was determined as the difference of the radioactivity accumulated in the synaptosomes in the presence and absence of 1 μM nomifensine.

The inhibition was determined as percent of controls and the IC_50 _values were calculated from at least four separate experiments, each conducted in duplicates, and the 95% confidence limits were calculated by non-linear regression analysis from the approximately linear part of the log concentration response curves.

### Statistical analysis

All c-Fos expression values are optical density measurements (arbitrary units) and are represented as mean ± S.E.M normalized to 100% of control. A one-way analysis of variance (ANOVA) was used. *Post-hoc *tests were performed using the Tukey test to compare significance between the individual groups. The significance was taken when *p*-values were less than 0.05.

## Results

### Effect of chronic pseudoephedrine treatment on the c-Fos acute response to pseudoephedrine in nucleus accumbens and striatum

**Figure 1:**
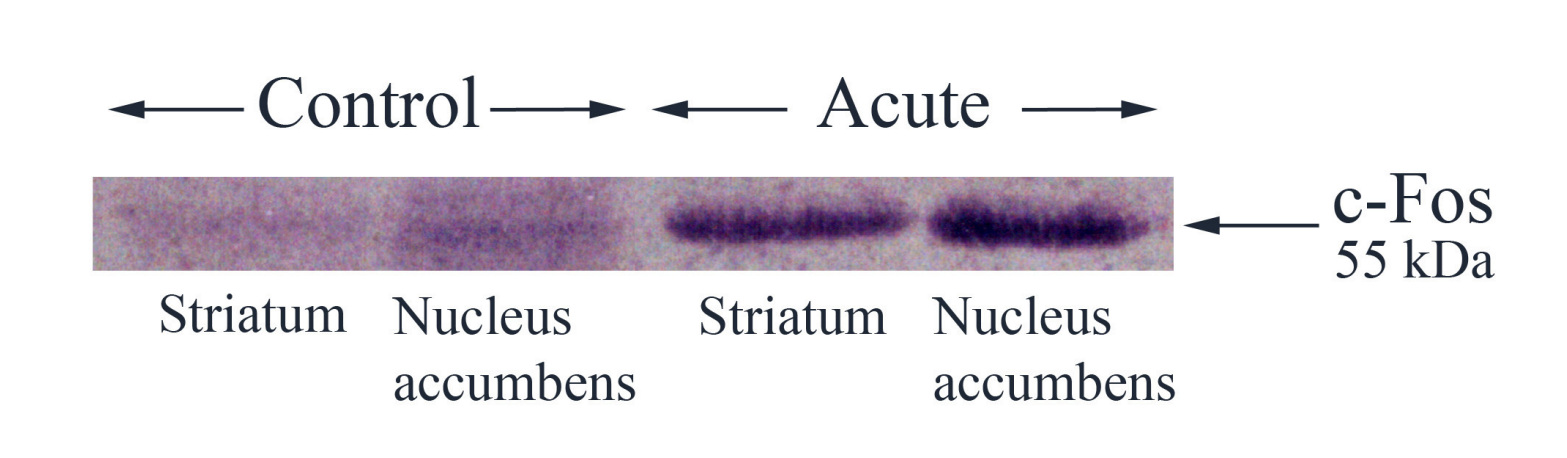
**Pseudoephedrine induced c-Fos expression**. Western blot analysis was used to measure the acute pseudoephedrine (40 mg/kg, i.p) induced c-Fos expression in striatum and nucleus accumbens. c-Fos protein was detected 1.5 h after saline and pseudoephedrine injection.

The relative c-Fos response in nucleus accumbens and striatum was measured by densitometry of immunostained bands in a Western blot assay. Pseudoephedrine (40 mg/kg, i.p.) induced an acute increase in c-Fos expression in the nucleus accumbens and striatum as compared with saline-treated rats (Fig. 1). The peak c-Fos response was reached at 1.5 hours after treatment. It was maintained for up to 30 further minutes and was just above control at 3 hours after treatment (results not shown). The bands represent the 55 KDa c-Fos peptide.

**Figure 2:**
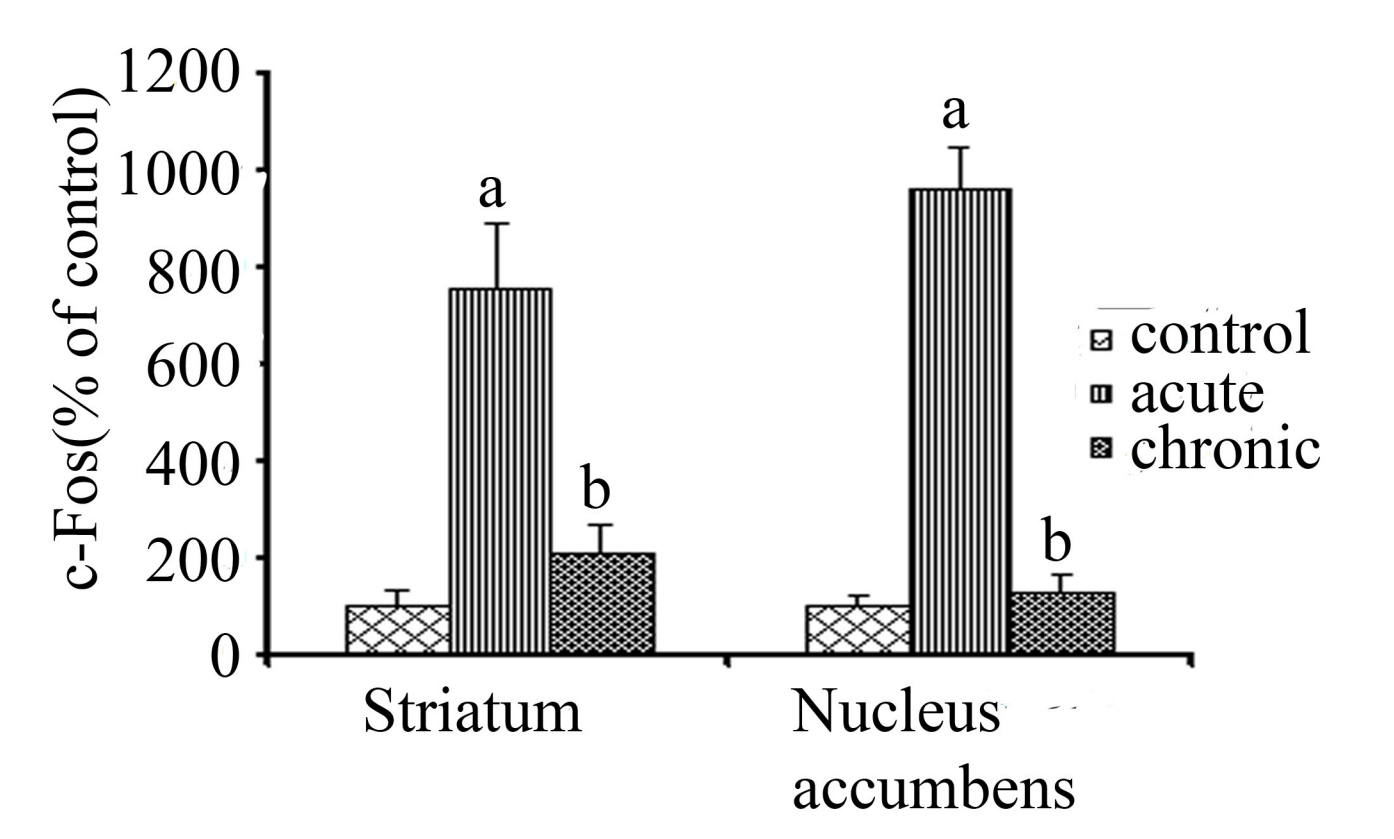
**Effect of chronic drug treatment on acute c-Fos response**. Western blot analysis was used to measure the acute pseudoephedrine induced c-Fos expression in striatum (n = 4) and nucleus accumbens (n = 3) of rats chronically treated with pseudoephedrine (8 day treatment of pseudoephedrine twice a day with an increasing dose at 25, 30, 35, 40 mg/kg i.p., on day 1, 2, 3 and 4–8 respectively, rats were killed 1.5 h after 40 mg/kg, i.p pseudoephedrine injection on day 9). The data (quantification of bands from western blots) is expressed as percentage of c-Fos value of control animals injected with saline. (A value of 100% indicates no difference from control) Values are mean ± S.E.M. 'a' indicates significantly different from control and chronic; 'b' indicates significantly different from acute with *p *< 0.05.

To determine effect of pseudoephedrine chronic treatment, rats were chronically injected with either saline or pseudoephedrine twice a day for 8 days and sacrificed 1.5 h after a final psuedoephedrine injection on day 9. Chronic exposure to pseudoephedrine significantly reduced (*p *< 0.05) the acute c-Fos response to pseudoephedrine in the nucleus accumbens and striatum as compared with animals chronically treated with saline (Fig. 2).

### Cross tolerance between pseudoephedrine and amphetamine

**Figure 3:**
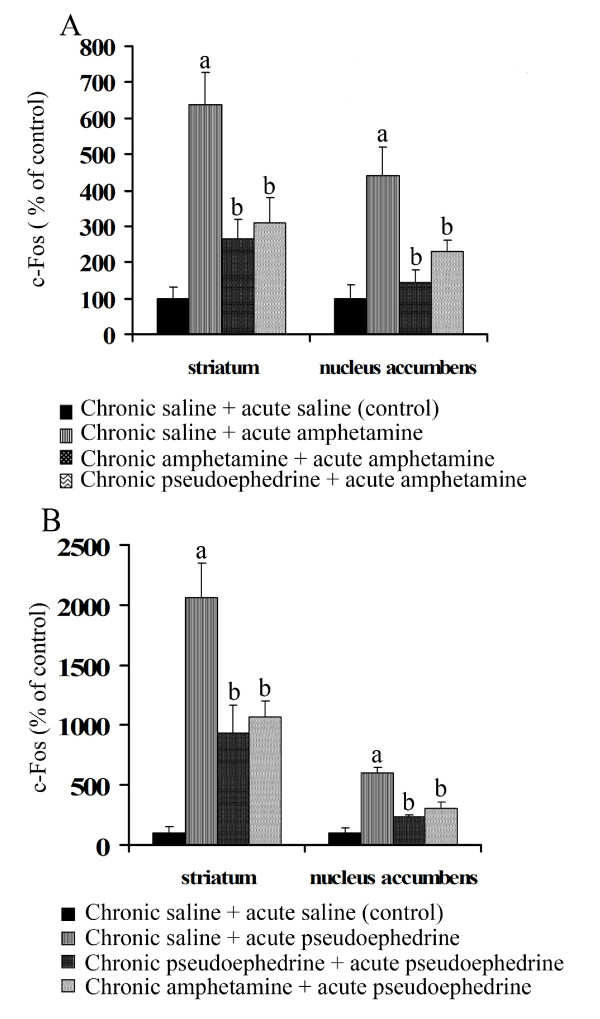
**Cross tolerance between amphetamine and pseudoephedrine**. Western blot analysis was used to measure the level of cross-tolerance between pseudoephedrine and amphetamine to drug induced c-Fos expression in striatum (n = 3) and nucleus accumbens (n = 3) in chronically treated rats. A: acute response to amphetamine in rats chronically treated with saline, amphetamine and pseudoephedrine (8 day treatment of drug twice a day with an increasing dose schedule; rats were killed 1.5 h after 40 mg/kg, i.p pseudoephedrine injection). There was no significant difference in the acute response to amphetamine between rats chronically treated with amphetamine or pseudoephedrine. B: acute response to pseudoephedrine in rats chronically treated with saline, pseudoephedrine and amphetamine (8 day treatment of drug twice a day with an increasing dose schedule, rats were killed 1.5 h after 40 mg/kg, i.p pseudoephedrine injection). There was no significant difference in the acute response to pseudoephedrine between rats treated with amphetamine or pseudoephedrine. The data are expressed as percentage of c-Fos value of control animals injected with saline. Values are mean ± S.E.M. a indicates significantly different from control and chronic; b indicates significantly different from acute, *p *< 0.05.

The following experiments were designed to test whether chronic treatment with pseudoephedrine also affected the acute response to other psychostimulants. Rats were chronically injected with either saline, amphetamine or psuedoephedrine for 8 days as described, and on day 9 they received a final injection of either pseudoephedrine or amphetamine respectively (i.e. pseudoephedrine treated rats received a final injection of amphetamine and vice versa) and sacrificed 1.5 h after the last injection. Measurements of c-Fos expression in the nucleus accumbens and striatum indicated that chronic treatment with either amphetamine or pseudoephedrine caused a reduction (*p *< 0.005) in the acute response to both drugs (Fig. 3) indicating that pseudoephedrine and amphetamine have cross tolerance and may use a common pathway to induce the expression of c-Fos protein.

### The effects of d-pseudoephedrine on [^3^H]dopamine uptake

**Figure 4:**
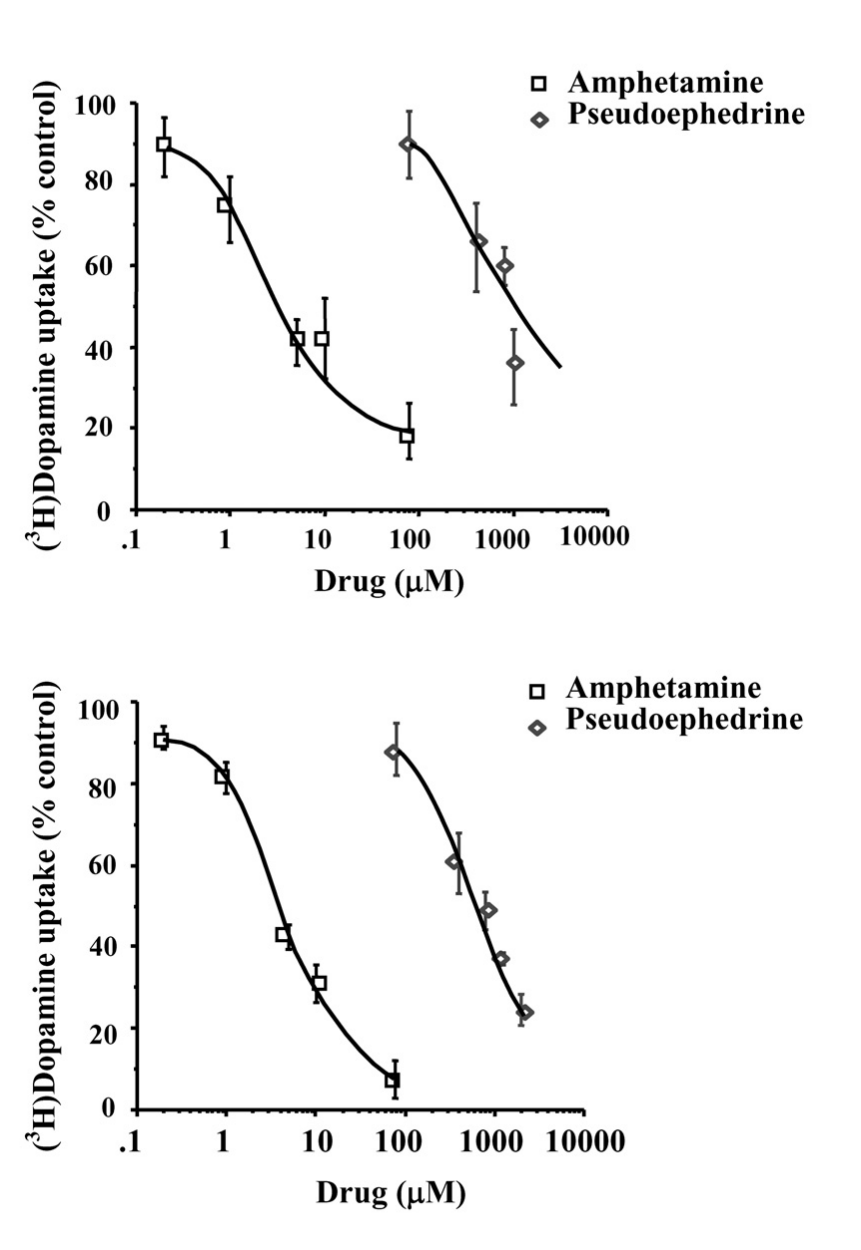
**[^3^H]dopamine uptake in rat brain synaptosomes**. Effect of *d*-amphetamine and *d*-pseudoephedrine on [^3^H]dopamine uptake in rat striatal (n = 8) and nucleus accumbens (n = 4) synaptosomes. IC_50 _is defined as the concentration of drug producing a 50 percent inhibition in 1 nM [^3^H]dopamine uptake. Values represent uptake as percentage of control. IC_50 _values are mean ± S.E.M. from duplicate samples in each independent experiment. The IC_50 _values of amphetamine in inhibiting [^3^H]dopamine uptake in striatum and nucleus accumbens are 4.28 ± 0.47 and 2.95 ± 0.42 μM, respectively, and of *d*-pseudoephedrine in inhibiting [^3^H]dopamine uptake in striatum and nucleus accumbens are 667 ± 126 and 530 ± 61 μM, respectively.

In order to investigate the mechanism of action of pseudoephedrine and to compare it to that of amphetamine, the effect of pseudoephedrine and amphetamine on [^3^H]dopamine uptake was assayed in synaptosomal preparations from rat brain areas. Both amphetamine and pseudoephedrine inhibited the uptake of [^3^H]dopamine into nucleus accumbens and striatal synaptosomes, with amphetamine being more potent than pseudoephedrine (Fig. 4). The amphetamine and pseudoephedrine curves were parallel to each other with the curve for pseudoephedrine inhibition of [^3^H]dopamine uptake shifted to the right. The IC_50 _values for amphetamine and pseudoephedrine inhibition reported here (see legend Fig. 4) indicate that amphetamine was 156 and 180 times more potent than pseudoephedrine at inhibiting [^3^H]dopamine uptake into striatal and nucleus accumbens synaptosomes, respectively.

## Discussion

Chronic injection of pseudoephedrine resulted in desensitisation of the acute pseudoephedrine induced c-Fos induction in the striatum and nucleus accumbens. It has previously been shown that chronic treatment with cocaine and amphetamine reduces the immediate-early-gene expression response to these drugs in specific brain areas [19-21]. Pseudoephedrine effects on c- Fos expression is thus similar to that of other psychostimulants both at the acute and chronic level. The c-Fos response to psychomotor stimulant drugs appears to be a direct response to the drug rather than to a general drug-related altered state. For example other studies have shown that there are no significant changes in c-Fos expression following withdrawal from chronic treatment of psychostimulants (22).

To further test the hypothesis that amphetamine and pseudoephedrine work through similar mechanisms, cross tolerance tests were carried out. The results indicate that in amphetamine and in pseudoephedrine chronically treated rats the c-Fos response in the striatum and nucleus accumbens following a final injection with pseudoephedrine and amphetamine respectively, was lower than in naïve animals. This result is consistent with our previous findings that pseudoephedrine has similar effects and mechanisms of action as amphetamine in terms of c-Fos expression [13] and internal behavioral cues [12]. In this work we have also analysed another reported effect of psychostimulants namely inhibition of [^3^H]dopamine uptake and report that both amphetamine and pseudoephedrine inhibit [^3^H]dopamine uptake. The role of dopamine in pseudoephedrine induced c-Fos response is further corroborated by the effect of the D1 receptor antagonist SCH23390 which as previously described [13] inhibited acute response to pseudoephedrine (results not shown).

## Conclusion

Our studies thus indicate that pseudoephedrine, notwithstanding the higher doses required, acts in manner indistinguishable from amphetamine. This may have sociological and medical implications as pseudoephedrine is a legal over-the-counter drug. Doses of pseudoephedrine in over-the-counter formulations vary between 60–120 mg/pill and a large amount of pills would need to be ingested to equate the doses injected into rats in this study. However, it is unknown whether the efficacy of the pseudoephedrine in humans is different from rats. An additional potential risk of pseudoephedrine is reinstatement of drug seeking behaviour in individuals that have overcome previous addiction to psychostimulants. It would be useful to determine whether pseudoephedrine could induce a reinstatement of drug seeking behaviour in rats that had first been chronically treated with amphetamine and that had then been allowed to extinguish their amphetamine seeking behaviour. Particular attention would need to be paid to the genetic background of the animals as it has been shown to affect relapse behaviour [23]. The data here reported and the additional approach described above would inform further studies in human subjects aimed at determining the potency of pseudoephedrine in humans to identify what doses may constitute a health, addiction or relapse risk.

## Authors' contributions

NR carried out all the animal treatment and western blot experiments and drafted the manuscript. WT carried out the synaptosome experiments and contributed to the draft of the manuscript. SOC devised the experiment plan for the western blots and finalized the manuscript. PG devised the experiment plan for the synaptosome work, critically reviewed the final manuscript preparation and provided overall coordination for the project.
